# The threat of antimalarial drug resistance

**DOI:** 10.1186/s40794-016-0027-8

**Published:** 2016-07-07

**Authors:** Borimas Hanboonkunupakarn, Nicholas J. White

**Affiliations:** 1 Department of Clinical Tropical Medicine, Faculty of Tropical Medicine, Mahidol University, 420/6 Ratchawithi Rd, Ratchathewi, Bangkok, 10400 Thailand; 2 Mahidol-Oxford Tropical Medicine Research Unit, Faculty of Tropical Medicine, Mahidol University, Bangkok, Thailand; 3 Centre for Tropical Medicine and Global Health, Nuffield Department of Medicine, University of Oxford, Oxford, UK

**Keywords:** Antimalarial drug resistance, Malaria, *Plasmodium falciparum*, Artemisinin, Chloroquine, Southeast Asia

## Abstract

The battle between man and malaria has continued for thousands of years. Antimalarial drugs are essential weapons to fight the disease, but their efficacy is threatened by drug resistance which continues to emerge creating a major obstacle to malaria control and jeopardizing renewed hopes for elimination. As 2016 is the first year under WHO Global Technical Strategy for Malaria 2016–2030, it is a good time to ponder the progress of both sides and plan for the future.

## Background

Malaria is an ancient disease that has existed for several thousand years (as evidenced from *Plasmodial* DNA in mummies [1]). It is the most important parasitic disease of man and it is still a major health problem in tropical countries [2]. The disease is caused by hemoprotozoa of the genus *Plasmodium*. These parasites are transmitted by the bites of infected female anopheline mosquitoes. Six malaria species are known to cause human malaria: *P. falciparum, P. vivax, P. ovale curtisi, P. ovale wallikeri, P. malariae,* and *P. knowlesi* [3]. According to the World Health Organisation (WHO), around 3.2 billion people are at risk of malaria infection and there were 214 million new cases of malaria in 2015. The African Region accounted for most cases of malaria (88 %), followed by the South-East Asia Region (10 %) and then the Eastern Mediterranean Region (2 %). In the same year, 438,000 deaths from malaria were estimated to have occurred: 90 % of these deaths occurred in the African Region [2]. However, the true numbers may be much higher due to health information problems in resource-poor settings [4, 5]. Previous study by Snow et al., using geographical and demographic data and epidemiologic models, suggested that global falciparum malaria could be up to 50 % higher than the number reported by WHO with 200 % higher estimates for areas outside Africa [6]. *P. falciparum* is responsible for the great majority of deaths from malaria. Most of the fatalities are in African children. The propensity for *P. falciparum* to cause severe disease was recognized by the great Italian malariologists within a few years of the discovery of the malaria parasite by Laveran in 1880. The dangerous characteristics of *P. falciparum* are both the parasite’s unique pathogenesis, causing microvascular obstruction in vital organs, and its ready ability to develop drug-resistance. While resistance to some antimalarials does occur in *P. vivax* [7–9], the other major species infecting humans, only in *P. falciparum* has resistance developed to all currently used antimalarials, including the artemisinin derivatives [10]. This review will summarize man’s recent struggles with malaria with an emphasis on drug resistance in *P. falciparum*.

## Main text

### The previous battles and lessons learnt

Malaria was once prevalent throughout most of the inhabited world including Europe and North America [11]. The evidence for man’s attempts to fight malaria can be dated back as far as 270 years BC in the ancient Chinese medical record Nei Jing or the Canon of Internal Medicine. The bark of Cinchona trees was known as a remedy for febrile illness among Peruvian people before it was introduced to Europe by the Jesuits in the 1630s. Although Guttman and Ehrlich demonstrated the antimalarial efficacy of methylene blue in 1891 [12], the development of synthetic antimalarials did not start until after the First World War with the discovery of plasmoquine (pamaquine) in 1924. Nevertheless, quinine remained the mainstay of antimalarial treatment. In the Second World War the loss of Java and thus 90 % of the world’s Cinchona supply to Axis forces stimulated an enormous research effort which rediscovered chloroquine (first synthesised in 1934 in Germany) and led to the antifolate drugs. Together with the discovery of the insecticidal properties of DDT (dichloro-diphenyl-trichloroethane) and its implementation in anopheline vector control, these drugs were the main weapons used in the Global Malaria Eradication campaign from 1955 to 1969. Malaria was eliminated from all Europe, most of the former USSR, North America, and many parts of South America, and Asia by the early 1970s [13]. However, misuse of antimalarial drugs, such as in the form of medicated salt [14], and widespread availability of low quality medicines [15] lead to the emergence of drug resistance. Chloroquine resistance in *Plasmodium falciparum* appeared almost at the same time in Thailand in Southeast Asia (SEA) in 1957 [16] and in Colombia in South America in 1959 [17]. Resistance spread in both areas [18]. Chloroquine resistance spread across SE Asia and India and arrived at the eastern seaboard of Africa in 1978 [19, 20]. In 14 years it then spread across the entire continent. The cost of chloroquine resistance in human lives was enormous. The number of malaria deaths rose through the 1980s to a peak in 2004 [21]. Chloroquine resistance had spread throughout the malaria endemic world, such that the distribution of chloroquine resistance was almost identical to that of *P. falciparum* by 1989 [22]. Recent genetic studies analyzing microsatellite markers surrounding the *Plasmodium falciparum* chloroquine resistance transporter gene (*pfcrt*) have confirmed that chloroquine resistance did spread from Asia to Africa [23]. Using the same technique it was shown that the main form of sulfadoxine-pyrimethamine resistance (triple 51, 59 and 108 dihydrofolate reductase (*dhfr)* gene mutations), previously believed to originate independently from numerous sources, also spread from SEA to Africa [24].

Mixed *P. falciparum* and *P. vivax* infections are common in areas of co-endemicity such as South [25] and SE Asia [26, 27]. The mixed infections can either manifest as a double infection at the beginning of treatment or as a sequential illness known as cryptic infection [25–27]. Cryptic *P. vivax* is believed to be a relapse from activated hepatic hypnozoites while cryptic *P. falciparum* is likely from erythrocytic asexual stages not killed by *P. vivax* treatment [28]. Improper treatments from failure to recognise mixed infections can lead to the development and spreading of drug resistance parasites [28].

The list of other drugs that *P. falciparum* has developed resistance to includes quinine [13], amodiaquine [29], mefloquine, halofantrine, lumefantrine, atovaquone, cycloguanil/chlorcycloguanil, sulphonamides and sulfones [30], piperaquine [31] and recently, artemisinin and its derivatives [32, 33]. More information about molecular markers of resistance and in vitro susceptibility antimalarials can be found in a recent review by Cui and coworkers [34] and in a website of Worldwide Antimalarial Resistance Network (WWARN) at http://www.wwarn.org/tracking-resistance.

### Artemisinin resistance: a terrible threat

Artemisinin (qinghaosu), a traditional Chinese medicine, is now the cornerstone of antimalarial therapeutics. It was considered so important that the 2015 Nobel Prize for Medicine or Physiology was awarded to Tu You You for her contribution to the discovery of this drug. Artemisinin and its derivatives provide the most rapid parasite clearance among known antimalarial drugs. It became a new hope as, during 1990s, resistance to available major antimalarial drugs such as chloroquine and sulfadoxine–pyrimethamine worsened across malaria endemic areas globally. Artemisinin-based monotherapies has been widely used in western Cambodia for more than 30 years [33]. Combination therapy with other antimalarials of a different class was proposed to delay the development of parasite resistance to artemisinin [35, 36]. The pairing of rapidly-eliminated artemisinin derivatives and the slowly-eliminated partner drugs also shortens the course of treatment compared to those of artemisinin monotherapies [37], thus increasing adherence. Three-day artemisinin-based combination therapies (ACTs) were introduced in the mid-1990s and shown to be highly effective and well-tolerated in treating falciparum malaria [37]. Finally since 2006, WHO has clearly recommended ACTs as the treatment of choice for uncomplicated *P. falciparum* malaria [38]. The relatively high cost of these drugs and the reluctance of donors to support them limited their deployment initially, but substantial subsidies particularly from the Global Fund to Fight AIDS, Tuberculosis and Malaria (GFATM) have led to a substantial increase in ACT availability in recent years. Despite the use of ACTs, delayed parasite clearance suggesting artemisinin resistance was reported in Thailand-Cambodia border in 2007 [32, 33]. The parasite clearance half-life was shown to be the best in-vivo phenotypic measure of resistance. Since then slowly clearing malaria infections, defined as having parasite clearance half-lives > 5 h, have been detected from southern Vietnam to central Myanmar [39]. This clearance has been linked with point mutations in the “propeller” region of a *P. falciparum* kelch protein gene on chromosome 13, also known as kelch 13 or K13 [40]. WWARN has provided an interactive map that shows the updated global prevalence of K13, The K13 Molecular Surveyor, at http://www.wwarn.org/molecular-surveyor-k13. Using the K13 marker it is now clear that artemisinin resistance has spread from the coast of Vietnam to the Eastern border of India [41, 42]. Declining artemisinin susceptibility also increases the selection pressure for the emergence of partner drug resistance due to a greater proportion of the infecting parasites remains [43]. While artemisinin resistance is associated with delayed parasite clearance or early treatment failure, long half-life partner drug resistance is associated late treatment failure [44]. Failing of partner drugs then could lead to higher clinical malaria, poor treatment outcome and increase the chance of spreading artemisinin-resistant parasites [45].

Why is it that resistance to all antimalarial drug classes seems to originate from a specific area of the world, i.e., Southeast Asia (SEA)?

### Southeast Asia: the epicenter of drug resistance

In Southeast Asia, factors such as human migration particularly amongst young men who travel extensively in forested areas and forest-dwelling mosquitoes may render conventional vector-control methods less effective, resulting in a greater reliance on drugs in malaria control. Several factors in malaria endemic regions of SEA are suitable for the selection and spread of resistant parasites. Being a low transmission area, many infections are in non-immune people who become symptomatic and seek medication [30, 46]. Lower levels of immunity and higher parasite burdens increase the probability that drug resistant mutants will survive [47, 48]. Antimalarial drugs including artemisinins have been widely available in the private sector. Problems about drug use in private sector include drug quality, high frequency of wrong dosing and use of monotherapy, and inadequate knowledge and training of service providers [49, 50]. In some areas, these problems are almost similar among public and private sector [51]. Counterfeit or falsified antimalarial drugs, often now containing a small amount of artemisinins, can be found in markets and are widely used [15]. Self-medication with inadequate doses or courses of treatment or low quality medicines cause poor treatment outcomes and lead to resistance [52]. Antimalarial drug resistance usually confers a fitness disadvantage upon malaria parasites, but parasites in the SE Asian region have been exposed to several different selective forces which has created a genetic background that may predispose to the emergence of resistance [53].

### The coming battle

It will be disastrous if artemisinin resistance reaches India and then Africa where most of the world’s malaria burden lies. Plans to control malaria now focus on the elimination of malaria in the greater Mekong subregion, the major source of drug-resistant parasites [54]. The methods recommended are strengthening of existing measures [54], which to date have not contained the spread of resistance nor the emergence of new foci in the region [41]. Containing resistance will become increasingly difficult as *P. falciparum* develops resistance to all antimalarial drug classes. Moreover, increasing numbers of international travelers [55] and improved regional transportation will facilitate spread of resistant parasites. Transmission blocking by using a single low-dose primaquine in every patient with *P. falciparum* infection in low transmission settings without the need for glucose-6-phosphate dehydrogenase (G6PD) testing is now recommended by WHO [56]. Recent pharmacokinetic studies show that co-administration of primaquine and some ACTs, namely dihydroartemisinin-piperaquine [57] and pyronaridine –artesunate [58], are well tolerated in healthy adult subjects. No significant pharmacokinetic alterations on ACTs, but increased plasma concentrations of primaquine were observed. Although use of primaquine in radical curative regimens requires G6PD testing, the single low dose of primaquine as a *P. falciparum* gametocytocide has proved safe and well tolerated in G6PD deficient individuals [59]. While the antimalarial drug pipeline is healthier than ever before, none of the new drugs in development will be generally available within 4 to 5 years or, more likely, a decade. Therefore, current containment plans must rely on existing medicines. Improved dosing regimens in special populations such as pregnant women and children whose pharmacokinetics and pharmacodynamics are different from general population [60, 61] will reduce the selective pressure to resistance, and new strategies such as triple antimalarial combinations may increase the useful therapeutic life of existing drugs.

The new drugs in development range from those with known chemical structures and/or mechanism of action with enhanced properties, such as tafenoquine (an 8-aminoquinoline), artefenomel (OZ439) (a synthetic peroxide), and P218 (an antifol); to new chemical classes such as KAE609 (a spiroindolone) and KAF156 (an imidazolopiperazine) [11]. The threat of potentially untreatable falciparum malaria is real, and will require radical action if malaria is to be eliminated before the available drugs become ineffective [30, 62, 63].

## Conclusions

After thousand years of man’s struggle with malaria, the disease is still an important health problem in the tropics. *P. falciparum*, the major cause of death from malaria, has developed resistance to all currently used antimalarials and after substantial improvements in control in recent years threatens a resurgence. The elimination of malaria in the greater Mekong subregion, which is the major source of drug-resistant parasites, is an urgent priority to avert this threat. New drugs and new strategies to use the existing drugs are urgently needed before untreatable falciparum malaria becomes a reality.

## Abbreviations

ACTs, Artemisinin-based combination therapies; BC, Before Christ; DDT, Dichloro-diphenyl-trichloroethane; *Dhfr*, Dihydrofolate reductase; DNA, Deoxyrionucleic acid; G6PD, Glucose-6-phosphate dehydrogenase; GFATM, Global Fund to Fight AIDS, Tuberculosis and Malaria; *Pfcrt*, *Plasmodium falciparum* chloroquine resistance transporter; SEA, Southeast Asia; USSR, The Union of Soviet Socialist Republics; WHO, World Health Organisation; WWARN, Worldwide Antimalarial Resistance Network.

## References

[1] Lalremruata A, Ball M, Bianucci R, Welte B, Nerlich AG, Kun JFJ (2013). Molecular identification of falciparum malaria and human tuberculosis co-infections in mummies from the fayum depression (lower Egypt). PLoS One.

[2] World Health Organisation (2015). World malaria report 2015.

[3] Sutherland CJ, Tanomsing N, Nolder D, Oguike M, Jennison C, Pukrittayakamee S (2010). Two nonrecombining sympatric forms of the human malaria parasite *Plasmodium ovale* occur globally. J Infect Dis.

[4] Chaulagai CN, Moyo CM, Koot J, Moyo HB, Sambakunsi TC, Khunga FM (2005). Design and implementation of a health management information system in Malawi: issues, innovations and results. Health Policy Plan.

[5] Mphatswe W, Mate KS, Bennett B, Ngidi H, Reddy J, Barker PM (2012). Improving public health information: a data quality intervention in KwaZulu-Natal. South Africa Bull World Health Organ.

[6] Snow RW, Guerra CA, Noor AM, Myint HY, Hay SI (2005). The global distribution of clinical episodes of *Plasmodium falciparum* malaria. Nature.

[7] Thanh PV, Hong NV, Van NV, Louisa M, Baird K, Xa NX (2015). Confirmed *Plasmodium vivax* resistance to chloroquine in central Vietnam. Antimicrob Agents Chemother.

[8] Añez A, Moscoso M, Laguna Á, Garnica C, Melgar V, Cuba M (2015). Resistance of infection by *Plasmodium vivax* to chloroquine in Bolivia. Malar J.

[9] Price RN, von Seidlein L, Valecha N, Nosten F, Baird JK, White NJ (2014). Global extent of chloroquine-resistant *Plasmodium vivax*: a systematic review and meta-analysis. Lancet Infect Dis.

[10] World Health Organisation (2010). Guidelines for the treatment of malaria.

[11] Flannery EL, Chatterjee AK, Winzeler EA (2013). Antimalarial drug discovery - approaches and progress towards new medicines. Nat Rev Microbiol.

[12] Guttmann P, Ehrlich P (1891). “Über die wirkung des methylenblau bei malaria” (on the effect of methylene blue on malaria). Berliner Klinische Wochenschrift.

[13] Bunnag D, Harinasuta T (1987). The current status of drug resistance in malaria. Int J Parasitol.

[14] D’Alessandro U, Buttiëns H (2001). History and importance of antimalarial drug resistance. Trop Med Int Health.

[15] Tabernero P, Fernández FM, Green M, Guerin PJ, Newton PN (2014). Mind the gaps--the epidemiology of poor-quality anti-malarials in the malarious world--analysis of the WorldWide Antimalarial Resistance Network database. Malar J.

[16] Harinasuta T, Migasen S, Bunnag D. Chloroquine resistance in Plasmodium falciparum in Thailand. UNESCO 1^st^ Regional Symposium on Scientific Knowledge of Tropical Parasites, Singapore: University of Singapore; 1962. p.148-153.

[17] Moore DV, Lanier JE (1961). Observations on two *Plasmodium falciparum* infections with an abnormal response to chloroquine. Am J Trop Med Hyg.

[18] World Health Organisation. 1973. Chemotherapy of Malaria and Resistance to Antimalarials. WHO Technical Report Series 529. World Health Organisation, Geneva. 4200089

[19] Fogh S, Jepsen S, Effersøe P (1979). Chloroquine-resistant *Plasmodium falciparum* malaria in Kenya. Trans R Soc Trop Med Hyg.

[20] Campbell CC, Chin W, Collins WE, Teutsch SM, Moss DM (1979). Chloroquine-resistant *Plasmodium falciparum* from east Africa: cultivation and drug sensitivity of the Tanzanian I/CDC strain from an American tourist. Lancet.

[21] Murray CJ, Rosenfeld LC, Lim SS, Andrews KG, Foreman KJ, Haring D (2012). Global malaria mortality between 1980 and 2010: a systematic analysis. Lancet.

[22] Wernsdorfer WH, Payne D (1991). The dynamics of drug resistance in *Plasmodium falciparum*. Pharmacol Ther.

[23] Wootton JC, Feng X, Ferdig MT, Cooper RA, Mu J, Baruch DI (2002). Genetic diversity and chloroquine selective sweeps in *Plasmodium falciparum*. Nature.

[24] Roper C, Pearce R, Nair S, Sharp B, Nosten F, Anderson T (2004). Intercontinental spread of pyrimethamine-resistant malaria. Science.

[25] Starzengruber P, Fuehrer HP, Ley B, Thriemer K, Swoboda P, Habler VE (2014). High prevalence of asymptomatic malaria in south-eastern Bangladesh. Malar J.

[26] Looareesuwan S, White NJ, Chittamas S, Bunnag D, Harinasuta T (1987). High rate of *Plasmodium vivax* relapse following treatment of falciparum malaria in Thailand. Lancet.

[27] Pukrittayakamee S, Imwong M, Looareesuwan S, White NJ (2004). Therapeutic responses to antimalarial and antibacterial drugs in vivax malaria. Acta Trop.

[28] Mayxay M, Pukrittayakamee S, Newton PN, White NJ (2004). Mixed-species malaria infections in humans. Trends Parasitol.

[29] Hall AP, Segal HE, Pearlman EJ, Phintuyothin P, Kosakal S (1975). Amodiaquine resistant falciparum malaria in Thailand. Am J Trop Med Hyg.

[30] White NJ (2004). Antimalarial drug resistance. J Clin Invest.

[31] Eastman RT, Dharia NV, Winzeler EA, Fidock DA (2011). Piperaquine resistance is associated with a copy number variation on chromosome 5 in drug-pressured *Plasmodium falciparum* parasites. Antimicrob Agents Chemother.

[32] Noedl H, Se Y, Schaecher K, Smith BL, Socheat D (2008). Fukuda MM; Artemisinin Resistance in Cambodia 1 (ARC1) Study Consortium. Evidence of artemisinin-resistant malaria in western Cambodia. N Engl J Med.

[33] Dondorp AM, Nosten F, Yi P, Das D, Phyo AP, Tarning J (2009). Artemisinin resistance in *Plasmodium falciparum* malaria. N Engl J Med.

[34] Cui L, Mharakurwa S, Ndiaye D, Rathod PK, Rosenthal PJ (2015). Antimalarial drug resistance: literature review and activities and findings of the ICEMR Network. Am J Trop Med Hyg.

[35] White NJ, Olliaro PL (1996). Strategies for the prevention of antimalarial drug resistance: rationale for combination chemotherapy for malaria. Parasitol Today.

[36] White NJ (1998). Preventing antimalarial drug resistance through combinations. Drug Resist Updat.

[37] White NJ, Hien TT, Nosten FH. A brief history of qinghaosu. Trends Parasitol. 2015 Nov 19. doi: 10.1016/j.pt.2015.10.010. 10.1016/j.pt.2015.10.010 PMC4674626 26776328

[38] World Health Organisation (2006). Guidelines for the treatment of malaria.

[39] Ashley EA, Dhorda M, Fairhurst RM, Amaratunga C, Lim P, Suon S (2014). Spread of artemisinin resistance in *Plasmodium falciparum* malaria. N Engl J Med.

[40] Ariey F, Witkowski B, Amaratunga C, Beghain J, Langlois AC, Khim N (2014). A molecular marker of artemisinin-resistant *Plasmodium falciparum* malaria. Nature.

[41] Takala-Harrison S, Jacob CG, Arze C, Cummings MP, Silva JC, Dondorp AM (2015). Independent emergence of artemisinin resistance mutations among *Plasmodium falciparum* in southeast Asia. J Infect Dis.

[42] Tun KM, Imwong M, Lwin KM, Win AA, Hlaing TM, Hlaing T (2015). Spread of artemisinin-resistant *Plasmodium falciparum* in Myanmar: a cross-sectional survey of the K13 molecular marker. Lancet Infect Dis.

[43] White NJ (2012). Counter perspective: artemisinin resistance: facts, fears, and fables. Am J Trop Med Hyg.

[44] Krishna S, Kremsner PG (2013). Antidogmatic approaches to artemisinin resistance: reappraisal as treatment failure with artemisinin combination therapy. Trends Parasitol.

[45] Slater HC, Griffin JT, Ghani AC, Okell LC (2016). Assessing the potential impact of artemisinin and partner drug resistance in sub-Saharan Africa. Malar J.

[46] Doolan DL, Dobaño C, Baird JK (2009). Acquired immunity to malaria. Clin Microbiol Rev.

[47] White N (1999). Antimalarial drug resistance and combination chemotherapy. Philos Trans R Soc Lond B Biol Sci.

[48] Hastings IM (2004). The origins of antimalarial drug resistance. Trends Parasitol.

[49] Cong LD, Yen PT, Nhu TV, Binh LN (1998). Use and quality of antimalarial drugs in the private sector in Viet Nam. Bull World Health Organ.

[50] Okeke TA, Uzochukwu BS, Okafor HU (2006). An in-depth study of patent medicine sellers’ perspectives on malaria in a rural Nigerian community. Malar J.

[51] Meremikwu M, Okomo U, Nwachukwu C, Oyo-Ita A, Eke-Njoku J, Okebe J (2007). Antimalarial drug prescribing practice in private and public health facilities in South-east Nigeria: a descriptive study. Malar J.

[52] Dondorp AM, Fairhurst RM, Slutsker L, Macarthur JR, Breman JG, Guerin PJ (2011). The threat of artemisinin-resistant malaria. N Engl J Med.

[53] Miotto O, Amato R, Ashley EA, Macinnis B, Almagro-Garcia J, Amaratunga C (2015). Genetic architecture of artemisinin-resistant *Plasmodium falciparum*. Nat Genet.

[54] World Health Organisation (2015). Strategy for malaria elimination in the greater Mekong subregion (2015–2030).

[55] World Tourism Organisation (2015). UNWTO annual report 2014.

[56] World Health Organisation (2012). Updated WHO policy recommendation (October 2012): single dose primaquine as a gametocytocide in *Plasmodium falciparum* malaria.

[57] Hanboonkunupakarn B, Ashley EA, Jittamala P, Tarning J, Pukrittayakamee S, Hanpithakpong W (2014). An open-label crossover study of primaquine and dihydroartemisinin-piperaquine pharmacokinetics in healthy adult Thai subjects. Antimicrob Agents Chemother.

[58] Jittamala P, Pukrittayakamee S, Ashley EA, Nosten F, Hanboonkunupakarn B, Lee SJ (2015). Pharmacokinetic interactions between primaquine and pyronaridine-artesunate in healthy adult Thai subjects. Antimicrob Agents Chemother.

[59] Bancone G, Chowwiwat N, Somsakchaicharoen R, Poodpanya L, Moo PK, Gornsawun G (2016). Single low dose primaquine (0.25 mg/kg) does not cause clinically significant haemolysis in G6PD deficient subjects. PLoS One.

[60] Ward SA, Sevene EJ, Hastings IM, Nosten F, Mcgready R (2007). Antimalarial drugs and pregnancy: safety, pharmacokinetics, and pharmacovigilance. Lancet Infect Dis.

[61] Tarning J, Zongo I, Somé FA, Rouamba N, Parikh S, Rosenthal PJ (2012). Population pharmacokinetics and pharmacodynamics of piperaquine in children with uncomplicated falciparum malaria. Clin Pharmacol Ther.

[62] Kain KC (1995). Chemotherapy and prevention of drug-resistant malaria. Wilderness Environ Med.

[63] Heppner DG, Ballou WR (1998). Malaria in 1998: advances in diagnosis, drugs and vaccine development. Curr Opin Infect Dis.

